# Urokinase versus Alteplase in Patients with Intermediate–High-Risk Pulmonary Embolism Treated with Ultrasound-Accelerated Endovascular Thrombolysis

**DOI:** 10.3390/jcm12124006

**Published:** 2023-06-12

**Authors:** Hani Al-Terki, Michael Gotzmann, Felix Mahfoud, Lucas Lauder, Andreas Mügge

**Affiliations:** 1 Cardiology and Rhythmology Department, University Hospital St. Josef Hospital–Bochum, Ruhr University–Bochum, Gudrunstraße 56, 44791 Bochum, Germany; 2 Klinik für Innere Medizin III, Kardiologie, Angiologie und Internistische Intensivmedizin, Universitätskliniken des Saarlandes, Saarland University, 66421 Homburg, Germany

**Keywords:** EKOS^TM^, pulmonary embolism, urokinase, USAT, alteplase

## Abstract

Background. Ultrasound-accelerated thrombolysis (USAT) is a safe and effective treatment for patients with intermediate–high-risk pulmonary embolism (PE). In all studies investigating USAT in the setting of PE, the recombinant tissue-plasminogen activator (rt-PA) alteplase or actilyse was used. Currently, there is a shortage of alteplase (Alteplase, Boehringer Ingelheim) in Europe. It is unknown whether the efficacy of urokinase (UK) is comparable with alteplase for USAT in patients with PE. Methods. Patients with intermediate–high-risk PE undergoing USAT with urokinase and alteplase were included in this study. One-to-one nearest neighbour matching was performed to account for baseline differences. We identified one patient treated with USAT and UK (*n* = 9) for each patient treated with USAT and alteplase (*n* = 9). Results. A total of 56 patients underwent USAT. The treatment was successful in all patients. The propensity score matched the identified nine pairs of patients. There were no statistically significant differences in the change in right ventricle-to-left ventricle (RV/LV) ratio (0.4 ± 0.3 versus 0.5 ± 0.4, *p* = 0.54), systolic pulmonary artery pressure (17.3 ± 8.0 versus 18.1 ± 8.1, *p* = 0.17), or improvement of RV function (5.8 ± 3.8 versus 5.1 ± 2.6, *p* = 1.0). The complication rates were comparable (11% in both groups, *p* = 0.55). There were no deaths in hospital or during 90 days in either group. Conclusions. In this case-matched comparison, the short-term clinical and echocardiographic outcomes showed comparable results between USAT–UK and USAT–rt-PA.

## 1. Introduction

Acute pulmonary embolism (PE) is the third leading cause of mortality in the Western world. Current European guidelines recommend risk stratification of patients with acute PE into high, intermediate-to-high, intermediate-to-low, and low risk categories [1]. In intermediate-to-high-risk PE patients, ultrasound-accelerated thrombolysis (USAT) may be an appropriate therapeutic option as compared with anticoagulation alone in terms of improving right ventricle (RV) function [2,3].

The thrombolytic substance used for USAT in PE patients is the recombinant tissue-type plasminogen activator (rt-PA). The OPTALYSE trial investigated the optimal dosage and duration of rt-PA delivery [4]. Due to a current supply shortage of rt-PA (Alteplase^®^, Boehringer Ingelheim, Ingelheim, Germany) in Europe, other thrombolytic agents have been tested for use with USAT, and urokinase (UK) was found to be a safe and efficient therapeutic modality [5].

However, a head-to-head comparison of the two thrombolytic agents has not been reported in the literature. The aim of this propensity-matched analysis was to directly compare USAT with rt-PA and USAT with UK in their ability to improve the right ventricle-to-left ventricle (RV/LV) ratio, systolic pulmonary artery pressure (sPAP), and right ventricle function.

## 2. Methods

### 2.1. Patient Population

A total of 56 consecutive patients with intermediate–high-risk PE at St. Josef Hospital in Bochum, Germany, underwent USAT therapy for acute PE between August 2021 and April 2023 using either rt-PA (*n* = 46) or UK (*n* = 10). All patients were reviewed by a local pulmonary embolism response team (PERT) and identified as suitable candidates for interventional PE therapy according to European guidelines. All patients provided written informed consent for the procedures. Only bilateral PEs were included in this study (9 UK patients and 40 rt-PA patients). The rt-PA group received 6 mg rt-PA over 6 h per catheter (in line with the third arm of the OPTALYSE study). The UK group received 300,000 IE over 10 h per catheter.

### 2.2. PE Diagnosis and Risk Stratification

In all cases, the diagnosis of PE was based on multi-slice computed tomography of the lung. After confirming the diagnosis, further risk stratification was performed according to the current guidelines into low, intermediate-to-low, intermediate-to-high, and high risks. Patients with elevated troponin T and NTproBNP, an sPESI score ≥ 1, and echocardiographic signs of right heart strain (elevated sPAP, reduced TAPSE, and RV/LV ratio ≥ 1) were classified as intermediate–high-risk and received anticoagulation therapy with heparin. Included in this study were patients with intermediate–high-risk PE with haemodynamic deterioration on anticoagulation.

### 2.3. Device Description

EKOS^TM^ (Boston scientific, Marlborough, MA, USA) is a USAT system combining conventional catheter-directed thrombolysis with high-frequency (2.2 MHz) and low-power (0.5 W per element) ultrasound, which enables thermal-mediated diffusion of the thrombolytics into the thrombus via a high concentration gradient. The ultrasound uses cavitation-induced microstreaming, which loosens the fibrin strands within the clot to induce mechanical breakdown of the clot. It also increases the surface area of the thrombus, causing more active plasminogen receptor sites to come into contact with the thrombolytic agent. The EKOS system can be inserted into the pulmonary artery via a 6 French sheath and consists of an infusion catheter, an ultrasound core wire, and a control unit.

### 2.4. Definition of Endpoints and Follow-Up

The primary endpoints were as follows:

- RV/LV ratio, tricuspid annular plane systolic excursion (TAPSE), and sPAP 24 h post-intervention;
- in-hospital mortality.

The secondary outcome measures were as follows:

- procedural complications;
- rate of bleeding according to the GUSTO scale;
- procedural time (min);
- amount of contrast (mL);
- fluoroscopy dose (Gy);
- fluoroscopy time (min).

### 2.5. Statistical Analysis

The categorical variables were expressed as counts (percentages), and the continuous variables were expressed as the mean (SD) or the median (interquartile range) and compared using the Student’s *t*-test and the Mann–Whitney U test, respectively. Propensity matching was performed to reduce the imbalance in the patients’ baseline characteristics and potential selection bias on endpoints for comparing rt-PA with UK in the USAT setting. A one-to-one nearest neighbour matching procedure was used to identify one control case treated with rt-PA (*n* = 9) for each case treated with UK (*n* = 9). Age, TAPSE, sPAP, PESI, and RV/LV ratio were included in the matching algorithm. All tests were two-sided, and *p*-values < 0.05 were considered significant. All statistical analyses were performed using SPSS (IBM SPSS Statistics 27 for Windows).

## 3. Results

### 3.1. Baseline Characteristics and Propensity Matching

The baseline characteristics of the entire cohort, including the matched cohort, are displayed in Table 1. After matching, there were no statistically relevant differences between the groups in baseline characteristics, echocardiographic, and laboratory parameters at admission.

### 3.2. Procedural Data and In-Hospital Events of the Matched Cohorts

Procedural details and in-hospital events are summarised in Table 2. The procedural times, fluoroscopy doses, and fluoroscopy times were similar. The ICU stay (1.3 ± 0.7 for USAT–UK versus 1.4 ± 0.7 for USAT–rt-PA) and the improvement in clinical and echocardiographic parameters were not statistically significant. One patient in the USAT–UK group had access-site bleeding rated as severe on the GUSTO scale and underwent surgical repair. In the USAT–rt-PA group, one patient had arteriovenous fistula, which was treated conservatively. Both patients received three packed red blood cells to a 3-point drop in haemoglobin. There were no in-hospital or 90-day mortalities in either group.

## 4. Discussion

Acute PE is the third leading cause of cardiovascular mortality [1]. The cornerstone of PE therapy remains anticoagulation. Patients with intermediate–high-risk PE have high mortality, even if they are normotensive [6]. In such cases, catheter-based techniques are of interest because of the increased risk of major bleeding or stroke associated with systemic thrombolysis and the theoretical limitation of systemic-infused thrombolytic agents. The latter may cause blood to be shunted toward the unobstructed pulmonary arteries rather than those with obstruction [7,8]. However, evidence concerning the safety and efficacy of USAT is still considerably less robust than that for systemic thrombolysis.

In a multicentre randomized, controlled trial, Kucher et al. investigated the safety and efficacy of USAT with 10 mg/catheter actilyse over 10 h in patients at intermediate risk PE. The study found that USAT was superior to anticoagulation with heparin alone in reversing RV dilatation at 24 h without increasing bleeding complications. The mean decrease in RV/LV ratio was 0.29 ± 0.18 [3].

The OPTALYSE study aimed to determine the lowest optimal rt-PA dose and delivery duration using USAT for the treatment of acute intermediate–high-risk PE. The patients were randomized into four groups. Similar to our collective, the third group in the OPTALYSE study received 6 mg per site over 6 h. This dose was associated with an improvement in RV/LV ratio of 0.42 ± 0.32 [4].

Ouriel et al. compared the efficacy of different thrombolytic agents in an in vitro venous model and reported that streptokinase was associated with the slowest rate of clot lysis. Urokinase was associated with an intermediate rate of clot lysis but appeared to have the greatest degree of fibrinolytic specificity. Although rt-PA was associated with improved efficacy early in perfusion, the differences between rt-PA and urokinase dissipated after 30 min. The study concluded that urokinase may be the most beneficial thrombolytic agent [9]. Urokinase with USAT was investigated recently in nine patients and it was found that 300,000 IE urokinase per lung over 10 h induced significant improvement of RV/LV ratio at 0.75 ± 0.29 [5]. However, the sample size of the study was too small to draw firm conclusions.

In our analysis, both thrombolytic agents were associated with a significant improvement in RV/LV ratio, sPAP, and TAPSE 24 h after therapy. Importantly, no significant differences could be observed regarding procedural, echocardiographic, clinical, or laboratory data regarding in-hospital events and mortality.

### 4.1. Procedural Data

Technical success, defined as successful placement of the catheters and initiation of lytic therapy, was achieved in all patients in both groups. Contrast agent use was minimal (0 mL in USAT-rt-PA versus 4 mL in USAT-UK, *p* = 0.23). The procedural time, fluoroscopy dose, and fluoroscopy time were not different (*p* = 0.12, 0.63, and 0.48, respectively). Unfortunately, we were unable to compare these findings with prior studies since they were not reported in other publications. The ICU and total in-hospital stays in both groups were similar but shorter as compared with other reports (3.2 ± 2.0) [10].

### 4.2. Echocardiographic and Clinical Data

The goal of reperfusion therapy in PE is to facilitate RV recovery, to increase systemic perfusion, to improve symptoms and survival, and to prevent chronic thromboembolic pulmonary hypertension [11]. In our analysis, we found a significant improvement in RV/LV ratio in both groups (0.4 ± 0.3 versus 0.5 ± 0.4, *p* = 0.64). Our outcomes are similar to those of ULTIMA and SEATTLE-II (0.3 and 0.42, respectively) and similar to those of the third arm of the OPTALYSE study (0.42 ± 0.32), which used the same lytic protocol as our study [2,3,4]. Both groups in our analysis showed a decrease in sPAP after treatment. These findings are slightly higher than those of ULTIMA and SEATTLE-II (9.8 mmHg and 14.3 mmHg, respectively), but very close to the results of Kaymaz et al. (17.8 mmHg) [12]. The mean change in sPAP in the third arm of the OPTALYSE study was at 12.5 ± 11.0 mmHg.

### 4.3. Clinical Events and Complications

According to the GUSTO bleeding scale, one severe bleeding event occurred in each group (11% of each group, *p* = 0.55), both of which were access-site complications. In the USAT–UK group, the bleeding occurred in a patient with massive thrombus load in the femoral vein on both sides, requiring the puncture to be performed high, making compression difficult. The patient received three packed red blood cells and underwent surgical repair. In the USAT–rt-PA group, the bleeding event occurred due to an arterio-venous fistula. The patient received two packed red blood cells. The bleeding stopped after manual compression. The use of ultrasound-guided punction may have prevented the bleeding in this case. Compared to the bleeding rate of SEATTLE-II, which was 10%, our outcome was very similar. However, compared to ULTIMA (0%), our complication rate was quite high [2,3]. Notably, in the ULTIMA trial, all patients were punctured under an ultrasound guide, and ULTIMA excluded patients with high bleeding risk, aged over 80 years, and patients who had undergone surgery within the previous 10 days. Our cohort was a real-world collective and included elderly patients as well as patients with high bleeding risk.

It is worth mentioning that since no bleeding scale has been validated in PE patients receiving thrombolytics, the criteria for minor and major bleeding differ between studies. In the SEATTLE II trial and in our study, GUSTO was used, whereas researchers in the Jena-Experience trial used the BARC (Bleeding Academic Research Consortium) score [2,13]. Both scores have been validated for acute coronary syndrome but not for PE.

### 4.4. Mortality

There were no in-hospital or 90-day deaths in either group. This is similar to the findings of ULTIMA (0% in-hospital mortality) and the third arm of OPTALYSE (0%), but slightly lower than that of the SEATTLE-II study (2.7%), Jena-Experience (3.9%), and Kaymaz et al. (5.7%) [2,3,4,12,13]. However, the sample size is underpowered to draw firm conclusions.

## 5. Limitations

The small sample size and the lack of randomization are limitations of the present study. We used a propensity score to achieve comparable groups of patients receiving the two thrombolytic agents included in the analysis. As this method generally requires large samples and because in such a sample size statistical non-significance does not necessarily indicate similarity, our results should be interpreted with caution. This is a real-world study that reflects the current clinical practices at a specialized, high-volume centre in Germany. Although the patients were treated using comparable procedural standards, minor bias could not be excluded. Large randomized studies are needed to validate our findings and to determine the optimum dose of urokinase needed to achieve the best outcomes.

## 6. Conclusions

In this case-matched comparison, the short-term clinical and echocardiographic outcomes showed comparable results between USAT–urokinase and USAT–rt-PA, suggesting that USAT with 300,000 IE urokinase over 10 h may be as safe and efficient as rt-PA.

## Figures and Tables

**Table 1:** Baseline characteristics of USAT patients treated with rt-PA and the matched population of USAT patients treated with UK.

	Entire Population		Matched Population (1:1)	
	USAT–UK	USAT–rt-PA	USAT–rt-PA	*p* Value
	(*n* = 9)	(*n* = 40)	(*n* = 9)	
Clinical characteristics and comorbidities				
Age, (years)	75.3 (8.5)	66.7 (13.8)	75.0 (8.6)	0.93
Female (*n* %)	4 (44%)	21 (52.5%)	4 (44%)	0.49
BMI (kg/m^2^)	28.9 (4.2)	29.0 (6.4)	26.9 (4.6)	0.74
Arterial hypertension	7 (77%)	20 (50%)	7 (77%)	0.46
Diabetes (*n*, %)	3 (33.3%)	4 (10%)	4 (44%)	0.93
Chronic heart failure (*n*, %)	3 (33.3)	4 (10%)	3 (33.3)	0.91
Dialysis (*n*, %)	0 (1.7%)	3 (7.5%)	0 (0%)	0.70
Coronary artery disease (*n*, %)	3 (33.3%)	3 (7.5%)	2 (22%)	0.84
Previous embolism (*n*, %)	4 (44.4%)	7 (17.5%)	5 (55.5%)	0.81
Cancer (*n*, %)	0 (0%)	7 (17.5%)	0 (0%)	0.72
Immobility (*n*, %)	3 (33.3%)	6 (15%)	4 (44.4%)	0.75
COPD (*n*, %)	4 (44.4%)	8 (20%)	3 (33.3%)	0.72
Echocardiographic characteristics baseline				
TAPSE (mm)	14.7 (3.2)	17 (5.0)	14.75 (3.5)	0.98
sPAP (mmHg)	47.5 (7.0)	48.6 (11.0)	52.4 (13.7)	0.49
RVEDd (mm)	46.5 (6.3)	45.7 (5.5)	44.3 (3.7)	0.48
LVEDd (mm)	39.3 (5.8)	24 (16)	38.6 (2.6)	0.77
RV/LV ratio	1.19 (0.11)	1.24 (0.22)	1.17 (0.09)	0.67
Vital parameters at admission				
Heart rate (bpm)	86.3 (6.4)	96.7 (16)	96.3 (11.4)	0.09
Systolic pressure (mmHg)	127.5 (19.2)	137 (25)	131.2 (34.9)	0.78
Respiratory rate (1/min)	24.4 (6.0)	20 (5)	17.7 (8.9)	0.2
Oxygen saturation (%)	91.4 (4.1)	92.6 (1.8%)	88.5 (7.5)	0.32
PESI score	96.4 (19.6)	97.3 (31.2)	92.1 (18.2)	0.45
Laboratory at admission				
Creatinine (mg/dL)	1.06 (0.28)	1.0 (0.35)	1.19 (0.44)	0.36
NTproBNP (pg/mL)	7526 (5762)	8724 (6520)	7941 (1273)	0.36
Troponin (pg/mL)	0.07 (0.05)	0.2 (0.36)	0.08 (0.02)	0.23
Haemoglobin (mg/dL)	12.9 (2.0)	12.9 (2.2)	12.3 (2.5)	0.54
Lactate (mmol/L)	2.8 (2.8)	2.1 (1.8)	4.1 (4.2)	0.45

Values are mean SD, *n* (%). *p* = comparison between USAT–UK and USAT–rt-PA. USAT = ultrasound-accelerated thrombolysis; rt-PA = recombinant tissue plasminogen activator; UK = urokinase; BMI = body mass index; COPD = chronic obstructive pulmonary disease; TAPSE = tricuspid annular plane systolic excursion; sPAP = systolic pulmonary artery pressure; LVEDd = left ventricular end-diastolic diameter; RVEDd = right ventricular end-diastolic diameter; RV/LV ratio = right ventricle/left ventricle ratio; NTproBNP = N-terminal pro-brain natriuretic peptide; PESI = pulmonary embolism severity index.

**Table 2:** Procedural characteristics and in-hospital events of patients treated with USAT–UK and of the matched population treated with USAT–rt-PA.

		Matched Population (1:1)	
	USAT–UK	USAT–rt-PA	*p* Value
	(*n* = 9)	(*n* = 9)	
Procedural data			
Procedural time (min)	11.7 (17.0)	10.2 (4.8)	0.12
Contrast agent (mL)	4 (10)	0 (0)	0.23
Fluoroscopy dose (Gy)	935 (678)	1118 (873)	0.63
Fluoroscopy time (min)	7.3 (2.4)	8.8 (5.0)	0.48
ICU stay (days)	1.3 (0.7)	1.4 (0.7)	0.84
Total stay (days)	5.5 (1.0)	6.3 (2.8)	0.37
Echocardiographic data after therapy			
TAPSE (mm)	20.6 (2.7)	20.6 (3.2)	
∆TAPSE (mm)	5.8 (3.8)	5.1 (2.6)	1.0
sPAP mmHg	35.11 (8.0)	28.5 (9.0)	
∆ sPAP (mmHg)	17.3 (8.0)	18.1 (8.1)	0.17
RVEDd (mm)	38.14 (4.1)	35.8 (4.8)	
∆RVEDd (mm)	8.4 (4.1)	8.5 (4.7)	0.37
LVEDd (mm)	40.0 (4.3)	40.0 (2.77)	
∆LVEDd (mm)	1.6 (2.4)	1.3 (2.1)	0.40
RV/LV ratio	0.75 (0.29)	0.67 (0.43)	
∆RV/LV ratio	0.4 (0.3)	0.5 (0.4)	0.64
Vital parameters after therapy			
Heart rate (bpm)	80.3 (10.6)	77.5 (4.3)	
∆heart rate (bpm)	9.6 (4.2)	8.8 (3.7)	0.49
Systolic blood pressure (mmHg)	132.6 (19.5)	128.2 (33.0)	
∆systolic blood pressure (mmHg)	5.1 (24.3)	3.0 (29.6)	0.73
Diastolic blood pressure (mmHg)	75.4 (11.9)	79.3 (14.2)	
∆diastolic blood pressure (mmHg)	6.1 (15.9)	5.3 (14.5)	0.54
Respiratory rate (1/min)	19.0 (4.1)	16.6 (3.3)	
∆respiratory rate (1/min)	2.3 (7.4)	1.12 (8.1)	0.21
Oxygen saturation (%)	91.4 (4.1)	88.5 (7.5)	
∆oxygen saturation (%)	3.5 (1.4)	4.2 (2.1)	0.32
Laboratory			
Creatinine (mg/dL)	1.03 (0.19)	1.2 (0.4)	
∆creatinine (mg/dL)	0.02 (0.6)	0.01 (0.2)	0.37
NTproBNP (pg/mL)	4615 (4669)	6721 (4768)	
∆NTproBNP (pg/mL)	2911 (1904)	2003 (5352)	0.26
Troponin T (pg/mL)	0.07 (0.05)	0.23 (0.39)	
∆Troponin T (pg/mL)	0.01 (0.04)	0.03 (0.05)	0.23
Clinical events			
Severe bleeding (GUSTO)	1 (11%)	1 (11%)	0.55
In-hospital mortality	0 (0)	0 (0%)	0.94
90-day mortality	0 (0.0%)	0 (0.0%)	0.28

Values are mean SD, *n* (%) USAT = ultrasound-accelerated thrombolysis; rt-PA = recombinant tissue plasminogen activator; UK = urokinase; ICU = intensive care unit; TAPSE = tricuspid annular plane systolic excursion; sPAP = systolic pulmonary artery pressure; LVEDd = left ventricular end-diastolic diameter; RVEDd = right ventricular end-diastolic diameter; RV/LV ratio = right ventricle/left ventricle ratio; NTproBNP = N-terminal pro-brain natriuretic peptide.

## Data Availability

Data is unavailable due to privacy or ethical restriction.

## Abbreviations

Body mass index	BMI
Chronic heart failure	CHF
Chronic obstructive pulmonary disease	COPD
Computed tomography	CT
Left ventricle	LV
Left ventricle end-diastolic diameter	LVEDd
N-terminal pro-brain natriuretic peptide	NTproBNP
Pulmonary embolism	PE
Recombinant tissue plasminogen activator	rt-PA
Right ventricle	RV
Right ventricle end-diastolic diameter	RVEDd
Standard deviation	SD
Systolic pulmonary artery pressure	sPAP
Tricuspid annular plane systolic excursion	TAPSE
Urokinase	UK
Ultrasound-accelerated thrombolysis	USAT
